# Supplementary material for: Epidemiology, Diagnosis and Management of Penile Cancer: Results from the Spanish National Registry of Penile Cancer
Source: Cancers (Basel). 2023 Jan 19;15(3):616. doi: 10.3390/cancers15030616 (PMC9913503; doi:10.3390/cancers15030616)
Supplement: Supplementary file 1 [file cancers-15-00616-s001.zip › cancers-2147378-supplementary.pdf]

Registro Nacional de Cáncer de Pene

Plantilla de ayuda para captura de datos

** es obligatorio*

Datos de Identificación

Identificador PIEM del Paciente*:

Su Identificador del Paciente*:

Remisor *Seleccionar solo una opción*

- ☐ Derivado por otro especialista
- ☐ Derivado por médico de familia
- ☐ Traído por familiar

Motivo de la consulta *Seleccionar solo una opción*

- ☐ Hallazgo visual
- ☐ Dolor
- ☐ Escozor
- ☐ Supuración

Total días de ingreso:

En el caso de que se trate de un paciente remitido desde diagnóstico primario en otro centro, por favor facilite los datos que permitan su identificación en el Centro de Origen:

Datos Epidemiológicos

Estado del prepucio

Presencia de Fimosis *Seleccionar solo una opción*

- ☐ No
- ☐ Si

Grado de fimosis *Seleccionar solo una opción*

- ☐ Leve
- ☐ Marcada

Postectomía-Circucisión *Seleccionar solo una opción*

- ☐ No
- ☐ Si

Edad circuncisión: ________

Hábitos

Higiene local *Seleccionar solo una opción*

- ☐ Correcta
- ☐ Deficiente

Tabaquismo *Seleccionar solo una opción*

- ☐ No fumador
- ☐ Esporádico
- ☐ < 1/2 paquete/día
- ☐ Entre 1/2 y 1 paquete/día
- ☐ Entre 1 y 2 paquetes/día

Hábitos sexuales

Promiscuidad *Seleccionar solo una opción*

- ☐ No
- ☐ Si

Edad inicio relaciones sexuales: ________

Uso de métodos barrera con parejas no estables *Seleccionar solo una opción*

- ☐ Siempre
- ☐ A veces

☐ Nunca

Antecedentes HPV en pareja *Seleccionar solo una opción*

☐ No
☐ Si

Antecedentes de lesiones peneanas asociadas *Seleccionar solo una opción*

☐ No
☐ Si

Datos de Diagnóstico Clínico

Diámetro:

Localización *Seleccionar solo una opción*

- ☐ Glande
- ☐ Mucosa prepucial
- ☐ Piel prepucial
- ☐ Surco coronal
- ☐ Piel del pene
- ☐ Uretra

Número de lesiones:

Morfología *Seleccionar solo una opción*

- ☐ Papilar
- ☐ Nodular
- ☐ Ulcerada
- ☐ Plana
- ☐ Verrucoso
- ☐ Necrótico
- ☐ Hemorrágico
- ☐ Otros

Contacta con *Seleccionar solo una opción*

- ☐ Submucosa
- ☐ Túnica albugínea
- ☐ Uretra
- ☐ Cuerpo Esponjoso
- ☐ Cuerpos Cavernosos

Pruebas de imagen *Seleccionar solo una opción*

- ☐ Ninguna
- ☐ Ecografía
- ☐ Resonancia

Biopsia previa *Seleccionar solo una opción*

- ☐ No
- ☐ Si

Fecha de biopsia previa:

Método de biopsia previa *Seleccionar solo una opción*

- [ ] Punch / Tru-cut
- [ ] Cuña
- [ ] Escisión lesión

Ganglios regionales *Seleccionar solo una opción*

- [ ] No palpables
- [ ] palpables

Consistencia *Seleccionar solo una opción*

- [ ] Duros
- [ ] Blandos

Movilidad *Seleccionar solo una opción*

- [ ] Móviles
- [ ] Fijos

Infiltración local *Seleccionar solo una opción*

- [ ] No
- [ ] Si

Edema en pierna *Seleccionar solo una opción*

- [ ] No
- [ ] Si

Edema en escroto *Seleccionar solo una opción*

- [ ] No
- [ ] Si

Ecografía Inguinal *Seleccionar solo una opción*

- [ ] No realizada
- [ ] Normal
- [ ] Sospechosa

Biopsia ganglionar ecodirigida *Seleccionar solo una opción*

- [ ] No
- [ ] Si

Fecha de biopsia ganglionar ecodirigida: ____________

TAC Ilio-Inguinal *Seleccionar solo una opción*

- [ ] No
- [ ] Si

RMN Ilio-Inguinal *Seleccionar solo una opción*

- [ ] No
- [ ] Si

PET/TAC *Seleccionar solo una opción*

- [ ] No
- [ ] Si

Biopsia selectiva Ganglio centinela *Seleccionar solo una opción*

- [ ] No
- [ ] Si

Localización Ganglios *Seleccionar solo una opción*

- [ ] No sospechosos
- [ ] Ingle derecha
- [ ] Ingle Izquierda
- [ ] Bilateral

Fecha de biopsia selectiva Ganglio centinela: ____________

Cronología de la biopsia selectiva de ganglio centinela *Seleccionar solo una opción*

- [ ] Previa al tratamiento definitivo del cáncer de pene
- [ ] Coetánea al tratamiento definitivo del cáncer de pene

Diámetro ganglionar mayor: ____________

Nº Ganglios sospechosos en Ingle derecha: ____________

Nº Ganglios sospechosos en Ingle izquierda: ____________

Método de biopsia ganglionar *Seleccionar solo una opción*

- [ ] No realizada
- [ ] Aguja fina
- [ ] Punch / Tru-cut
- [ ] Cuña
- [ ] Escisión ganglio

Fecha Biopsia Ganglio Inguinal: ____________

Resultados y Estadificación Clínica

Determinación de DNA de HPV en la lesión *Seleccionar solo una opción*

- [ ] No
- [ ] Si

Tipo de HPV:

Resultado de la biopsia diagnóstica sobre tumor primario *Seleccionar solo una opción*

- [ ] Cáncer de pene
- [ ] Otros tumores malignos
- [ ] Lesión benigna
- [ ] Material insuficiente

Resultado de la biopsia diagnóstica sobre ganglio unguinal por biopsia percutánea ecodirigida *Seleccionar solo una opción*

- [ ] Cáncer de pene
- [ ] Otros tumores malignos
- [ ] Lesión benigna
- [ ] Material insuficiente

Estadio T clínico *Seleccionar solo una opción*

- [ ] TX
- [ ] T0
- [ ] Tis
- [ ] Ta
- [ ] T1a
- [ ] T1b
- [ ] T2 (cuerpo esponjoso/glande)
- [ ] T2 (cuerpo cavernoso)
- [ ] T3
- [ ] T4

Estadio N clínico *Seleccionar solo una opción*

- [ ] NX
- [ ] N0
- [ ] N1
- [ ] N2
- [ ] N3

Resultado de la biopsia diagnóstica sobre ganglio centinela *Seleccionar solo una opción*

- [ ] Cáncer de pene
- [ ] Otros tumores malignos
- [ ] Lesión benigna
- [ ] Material insuficiente

Infiltración de Cápsula clínica *Seleccionar solo una opción*

- [ ] No
- [ ] Si

Estadio M clínico *Seleccionar solo una opción*

- [ ] M0
- [ ] M1

Ubicación M clínico: ______

Grado Histológico (clínico) *Seleccionar solo una opción*

- [ ] GX
- [ ] G1
- [ ] G2
- [ ] G3-4

Tipo Histológico clínico *Seleccionar solo una opción*

- [ ] Carcinoma de células escamosas
- [ ] Carcinoma Neuroendocrino Primario
- [ ] Enfermedad de Paget
- [ ] Carcinoma de anejos
- [ ] Carcinoma de células claras
- [ ] Carcinoma Indeterminado
- [ ] Melanoma
- [ ] Metastásico
- [ ] Otros

Tipo de Carcinoma de Células Escamosas clínico *Seleccionar solo una opción*

- [ ] Clásico
- [ ] Basaloide
- [ ] Condilomatoso
- [ ] Papilar
- [ ] Verrucoso
- [ ] Sarcomatoide
- [ ] Mixto
- [ ] Adenoescamoso

Patrón de crecimiento clínico *Seleccionar solo una opción*

- [ ] Superficial
- [ ] Nodular/Fase vertical no infiltrante
- [ ] Infiltrativo

Profundida de la lesión: ____

Invasión Perineural clínico *Seleccionar solo una opción*

- [ ] No
- [ ] Si

Invasión Linfovascular clínico *Seleccionar solo una opción*

- [ ] No
- [ ] Si

Datos de Tratamiento

Tratamiento Lesión Primaria

Fecha tratamiento Lesión primaria:

Terapia Tópica *Seleccionar solo una opción*

- ☐ No realizada
- ☐ 5-Fluoracilo
- ☐ Imiquimod al 5%
- ☐ Otros

Terapia Láser *Seleccionar solo una opción*

- ☐ No realizada
- ☐ Láser CO2
- ☐ Láser Nd:Yag
- ☐ Otro láser

Cirugía *Seleccionar solo una opción*

- ☐ No realizada
- ☐ Cirugía micrográfica de Mohs
- ☐ Escisión en cuña
- ☐ Glandectomía
- ☐ Penectomía parcial
- ☐ Penectomía total

Circunsión concomitante *Seleccionar solo una opción*

- ☐ Ya realizada
- ☐ No realizada
- ☐ Realizada concomitantemente

Radioterapia *Seleccionar solo una opción*

- ☐ No realizada
- ☐ Neoadyuvante
- ☐ Adyuvante
- ☐ Neo y Adyuvante

Tipo de Radioterapia *Seleccionar solo una opción*

- ☐ Braquiterapia
- ☐ Radioterapia externa
- ☐ Braquiterapia y Radioterapia externa

Dosis:

Quimioterapia en relación a tratamiento de la lesión primaria *Seleccionar solo una opción*

- [ ] No realizada
- [ ] Neoadyuvante
- [ ] Adyuvante
- [ ] Neo y Adyuvante

Protocolo Quimioterapia:

Manejo Ganglionar

Fecha tratamiento Ganglios Inguinales:

Vigilancia *Seleccionar solo una opción*

- [ ] No
- [ ] Si

Antibioterapia previa *Seleccionar solo una opción*

- [ ] No
- [ ] Si

Linfadenectomía Inguinal *Seleccionar solo una opción*

- [ ] No realizada
- [ ] Izquierda
- [ ] Derecha
- [ ] Bilateral

Linfadenectomía inguinal; extensión del especímen *Seleccionar solo una opción*

- [ ] Radical
- [ ] Modificada

Tipo de Linfadenectomía Inguinal *Seleccionar solo una opción*

- [ ] Abierta
- [ ] Laparoscópica
- [ ] Robótica

Nº Ganglios extraídos:

Nº Ganglios afectados:

Manejo ganglios pélvicos

Fecha tratamiento Ganglios Pélvicos:

Linfadenectomía Pélvica *Seleccionar solo una opción*

- ☐ No realizada
- ☐ Izquierda
- ☐ Derecha
- ☐ Bilateral

Vigilancia *Seleccionar solo una opción*

- ☐ No
- ☐ Si

Tipo de linfadectomía pélvica *Seleccionar solo una opción*

- ☐ Abierta
- ☐ Laparoscópica
- ☐ Robótica

Nº Ganglios extraídos:

Nº Ganglios afectados:

Tratamiento de la afectación ganglionar

Quimioterapia afectación gangionar *Seleccionar solo una opción*

- ☐ No realizada
- ☐ Neoadyuvante
- ☐ Adyuvante
- ☐ Neo y Adyuvante

Protocolo Quimioterapia:

Número de ciclos:

Radioterapia Ganglionar *Seleccionar solo una opción*

- ☐ No
- ☐ Si

Tipo de Radioterapia *Seleccionar solo una opción*

- ☐ No realizada
- ☐ Neoadyuvante
- ☐ Adyuvante
- ☐ Neo y Adyuvante

Campo Radioterapia Ganglionar:

Dosis de Radioterapia Ganglionar:

Resultados y Estadificación Patológica

Estadio T patológico *Seleccionar solo una opción*

- [ ] pTX
- [ ] pT0
- [ ] pTis
- [ ] pTa
- [ ] pT1a
- [ ] pT1b
- [ ] pT2 (cuerpo esponjoso/glande)
- [ ] pT2 (cuerpo cavernoso)
- [ ] pT3
- [ ] pT4

Estadio N patológico *Seleccionar solo una opción*

- [ ] pNX
- [ ] pN0
- [ ] pN1
- [ ] pN2
- [ ] pN3

Infiltración de Cápsula patológica *Seleccionar solo una opción*

- [ ] No
- [ ] Si

Estadio M patológico *Seleccionar solo una opción*

- [ ] pM0
- [ ] pM1

Ubicación M patológico: ____________

Grado Histológico (patológico) *Seleccionar solo una opción*

- [ ] GX
- [ ] G1
- [ ] G2
- [ ] G3-4

Tipo Histológico patológico *Seleccionar solo una opción*

- [ ] Carcinoma de células escamosas
- [ ] Carcinoma Neuroendocrino Primario
- [ ] Enfermedad de Paget

- [ ] Carcinoma de anejos
- [ ] Carcinoma de células claras
- [ ] Carcinoma Indeterminado
- [ ] Melanoma
- [ ] Metastásico

Tipo de Carcinoma de Células Escamosas patológico *Seleccionar solo una opción*

- [ ] Clásico
- [ ] Basaloide
- [ ] Condilomatoso
- [ ] Papilar
- [ ] Verrucoso
- [ ] Sarcomatoide
- [ ] Mixto
- [ ] Adenoescamoso

Patrón de crecimiento patológico *Seleccionar solo una opción*

- [ ] Superficial
- [ ] Nodular/Fase vertical no infiltrante
- [ ] Infiltrativo

Profundidad de la lesión: ______

Invasión Perineural patológico *Seleccionar solo una opción*

- [ ] No
- [ ] Si

Invasión Linfovascular patológico *Seleccionar solo una opción*

- [ ] No
- [ ] Si

Márgenes patológicos *Seleccionar solo una opción*

- [ ] Libres
- [ ] Afectados

Complicaciones

Síntomas

Linforrea persistente *Seleccionar solo una opción*

- [ ] No
- [ ] Si

Días de linforrea persistente:

Linfocele *Seleccionar solo una opción*

- [ ] No
- [ ] Si

Infección del linfocele *Seleccionar solo una opción*

- [ ] No
- [ ] Si

Necesidad de drenaje linfocele *Seleccionar solo una opción*

- [ ] No
- [ ] Si

Edema escrotal *Seleccionar solo una opción*

- [ ] No
- [ ] Si

Edema Miembro Inferior *Seleccionar solo una opción*

- [ ] No
- [ ] Si

Necrosis cutánea *Seleccionar solo una opción*

- [ ] No
- [ ] Si

Infección de la herida *Seleccionar solo una opción*

- [ ] No
- [ ] Si

Tolerancia

Tolerancia de la Radioterapia:

Toxicidad de la Quimioterapia:
